# Adaptation of the Start-Growth-Time Method for High-Throughput Biofilm Quantification

**DOI:** 10.3389/fmicb.2021.631248

**Published:** 2021-08-26

**Authors:** Lara Thieme, Anita Hartung, Kristina Tramm, Julia Graf, Riccardo Spott, Oliwia Makarewicz, Mathias W. Pletz

**Affiliations:** ^1^Institute for Infectious Diseases and Infection Control, Jena University Hospital/Friedrich-Schiller-University, Jena, Germany; ^2^Leibniz Center for Photonics in Infection Research, Jena University Hospital/Friedrich-Schiller-University, Jena, Germany

**Keywords:** biofilms, high-throughput biofilm susceptibility testing, BBC, VISA hypothesis, dalbavancin

## Abstract

Colony forming unit (CFU) determination by agar plating is still regarded as the gold standard for biofilm quantification despite being time- and resource-consuming. Here, we propose an adaption of the high-throughput Start-Growth-Time (SGT) method from planktonic to biofilm analysis, which indirectly quantifies CFU/mL numbers by evaluating regrowth curves of detached biofilms. For validation, the effect of dalbavancin, rifampicin and gentamicin against mature biofilms of *Staphylococcus aureus* and *Enterococcus faecium* was measured by accessing different features of the viability status of the cell, i.e., the cultivability (conventional agar plating), growth behavior (SGT) and metabolic activity (resazurin assay). SGT correlated well with the resazurin assay for all tested antibiotics, but only for gentamicin and rifampicin with conventional agar plating. Dalbavancin treatment-derived growth curves showed a compared to untreated controls significantly slower increase with reduced cell doubling times and reduced metabolic rate, but no change in CFU numbers was observed by conventional agar plating. Here, unspecific binding of dalbavancin to the biofilm interfered with the SGT methodology since the renewed release of dalbavancin during detachment of the biofilms led to an unintended antimicrobial effect. The application of the SGT method for anti-biofilm testing is therefore not suited for antibiotics which stick to the biofilm and/or to the bacterial cell wall. Importantly, the same applies for the well-established resazurin method for anti-biofilm testing. However, for antibiotics which do not bind to the biofilm as seen for gentamicin and rifampicin, the SGT method presents a much less labor-intensive method suited for high-throughput screening of anti-biofilm compounds.

## Introduction

Microbial communities that are surrounded by a matrix of extracellular polymeric substance are commonly defined as biofilms (Hall-Stoodley et al., 2012). Biofilms represent the preferred life-form of pathogenic bacteria, following that they play a key role in many infectious diseases such as endocarditis, osteomyelitis, urinary tract infections and joint and soft tissue infections (Flemming et al., 2016). Increased antibiotic tolerance and/or resistance are one of the major hallmarks of biofilm-associated infections (Stewart, 2002). Since biofilm-embedded bacteria are usually genetically susceptible but phenotypically resistant, biofilm susceptibility is not predictable by the study of planktonic cells. Currently, biofilm-associated antibiotic tolerance is not addressed by microbiological routine diagnostics and treatment of biofilm-associated infections is guided by planktonic MIC testing, resulting in therapy failure and relapses. A multitude of biofilm susceptibility testing methods has been suggested, but none has so far reached a balance between the simplicity of a high-throughput method and the complex representation of the *in vivo* biofilm situation (Azeredo et al., 2017; Coenye et al., 2018; Magana et al., 2018). Further, standardization of the existing methods, including consistent interpretation of results and according recommendations, is lacking (Cruz et al., 2018; Thieme et al., 2019).

Colony forming unit (CFU) determination by agar plating is still regarded as the gold standard among bacterial quantification methods, including biofilms (Azeredo et al., 2017). Since agar-plating is time- and resource-consuming, we aimed to indirectly depict CFU/mL numbers by a culture-based method. Therefore, we adapted the recently published Start-Growth-Time (SGT) method to anti-biofilm testing, which allows a rapid quantification of the absolute and relative number of live cells in a high throughput manner (Hazan et al., 2012). The principle is comparable to the methodology of quantitative PCR calculations. After treatment, the biofilms are dispersed, diluted and regrown under continuous measurement of the optical density (OD) to obtain growth curves. The lag-phases of these growth curves are proportional to the number of cells in the dispersed biofilms, i.e., the more efficient the anti-biofilm treatment, the less CFU/mL, the longer the lag-phase. The SGT of each sample is defined as the time required to reach a defined OD threshold within the early to midst log-phase of the culture (Hazan et al., 2012). The growth delay of the treated growth curves, i.e., the respective SGTs, can be correlated to the quantity of CFU/mL reduction in comparison to the untreated control by CFU-SGT-standard curves of the same untreated strain. This minimizes the standard agar-plating procedure to a limited number of plates while simultaneously allowing the indirect measurement of CFU reduction of up to 96 samples.

To compare the CFU reduction results obtained with the novel SGT method with those obtained by conventional agar plating and resazurin metabolic assay, we treated mature biofilms of *Staphylococcus aureus* and *Enterococcus faecium*—which are one of the main pathogens causing biofilm-associated infections such as endocarditis and prosthetic joint infections—with serial concentrations of the antibiotics dalbavancin, rifampicin and gentamicin. Dalbavancin is a novel lipoglycopeptide with so far limited knowledge on biofilm eradication capability (Neudorfer et al., 2018), while the bactericidal antibiotics gentamicin and rifampicin were shown to exhibit anti-biofilm activity against Gram-positive biofilms (Sandoe et al., 2006; Coraça-Huber et al., 2012; Zimmerli and Sendi, 2019). The methods applied for biofilm quantification accessed different features of the viability status of the cells, i.e., the cultivability (CFU agar plating), growth behavior (SGT) and the metabolic activity (resazurin assay).

## Materials and Methods

### Bacterial Strains and Antibiotics

The clinical *S. aureus* isolates (MSSA: SA4733 and SA1642, MRSA: SA4002) and *E. faecium* isolates (VSE: EF24498 and EF12713, VRE: EF17129) were obtained from the Institute of Medical Microbiology at Jena University Hospital, Germany. Test solutions of dalbavancin (Correvio GmbH, Bielefeld, Germany), rifampicin (Sigma Aldrich, St. Louis, United States) and gentamicin (TCI Europe, Zwijndrecht, Belgium) were prepared freshly for each experiment.

### Biofilm Formation and Antibiotic Treatment

For biofilm maturation, 200 μL of 0.5 McFarland bacterial cultures were incubated in 96-well microtiter plates for 48 h at 37°C in a humidified chamber. *S. aureus* isolates were grown in Müller-Hinton (MH) broth and *E. faecium* isolates in Todd-Hewitt (TH) broth (both obtained from Sigma Aldrich, St. Louis, United States). For antibiotic treatment, the supernatants with planktonic cells were removed carefully, antibiotic solution with selected concentrations were prepared in the respective media and 200 μL per well were added to the biofilm. Pure media was used as growth control. Biofilms were incubated for additional 24 h at 37°C. Then, supernatant was removed and biofilms were washed two times with 0.9% NaCl before analyzing the biofilm reduction with the different methods. Each experiment was done in triplicates. To compare the different quantification methods, the biofilm bactericidal concentration (BBC), which is the lowest concentration of an antibiotic reducing 99.9% of biofilm-embedded bacteria (3 log_10_ reduction in CFU/mL) compared to the growth control (Macià et al., 2014), was determined for each antibiotic and strain by each method.

### CFU Determination by Agar Plating

The washed biofilms were scraped off the wells via vigorous scraping with a 100 μL pipette (i.e., the pipette tip was moved with pressure in all directions of the well’s bottom) and pipetting up and down, and resuspended in fresh MH or TH broth. For serial 10-fold dilution, 50 μL of each biofilm were transferred in MH or TH broth and vortexed to homogenize the biofilm debris. From selected dilutions, 100 μL were plated on MH or TH agar plates. After incubation of 18 h, colony forming units (CFU_*AGAR*_) were counted and bactericidal effects were calculated in relation to the untreated control biofilms.

### Resazurin Assay

Biofilm analysis by resazurin metabolism was adopted from Van den Driessche et al. (2014). Hundred microliter of a 10^–2^ dilution (taken from the previously prepared dilution series for agar plating) were added to a new 96-well microtiter plate and mixed with 10 μL alamarBLUE cell viability reagent (Thermo Fisher Scientific, Dreieich, Germany). Fluorescence was measured every 10 min for 18 h with a microtiter plate reader (Infinite M200pro, Tecan, Switzerland). Measurements were done at 37°C and with subsequently shaking for optimized growth conditions. For each isolate, a dilution series of a control biofilm was simultaneously measured to create a resazurin standard curve. The time to reach maximum fluorescence (t_*max*_) was determined for each biofilm. The t_*max*_ and the CFU_*AGAR*_ of the tested dilution series were correlated by linear regression to set-up a standard curve and to determine the detection limit for each strain. From this standard curve, the CFU_*RESA*_ of the treated and untreated biofilms were calculated by the corresponding t_*max*_ and subtracted from each other (ΔCFU_*RESA*_).

### Start-Growth-Time Method

Biofilms were washed twice with 0.9% NaCl, resuspended in fresh media and dispersed via vigorous scraping with a 100 μL pipette (Cruz et al., 2018). Dispersed biofilms were diluted 1:10 in fresh media and regrown in 96-well microtiter plates. The optical density was measured every 10 min at 600 nm for 18 h at 37°C in a microtiter plate reader under shaking conditions (Sunrise, Tecan, Switzerland). The SGT of each sample was defined as the time required to reach an OD_600 *nm*_ threshold that was set at the start to midst of the logarithmic phase, depending on the resulting growth curves. For the relative comparison of treated and untreated samples, the absolute size of the OD_600 *nm*_ threshold was not decisive but the unification for all samples. SGT values were normalized to the controls by the formula ΔSGT = SGT_*treated*_ − SGT_*control*_. To assess the linearity between SGT and CFU_*AGAR*_ values and thereby the detection limit for each strain, a standard curve was performed on every run. Therefore, SGTs of a serial diluted control biofilm and, in parallel, CFU_*AGAR*_ counts were determined. The CFU_*SGT*_ reduction due to antibiotic treatment was calculated by the standard curve log_10_ CFU_*AGAR*_ (x-axis) versus SGT (y-axis), whereby the SGT time span correlating to 1 log_10_ CFU difference was given by the slope of the linear regression. The resulting log_10_ CFU reduction was calculated by Δlog_10_ CFU_*SGT*_ = ΔSGT/slope.

## Results

**FIGURE 1:**
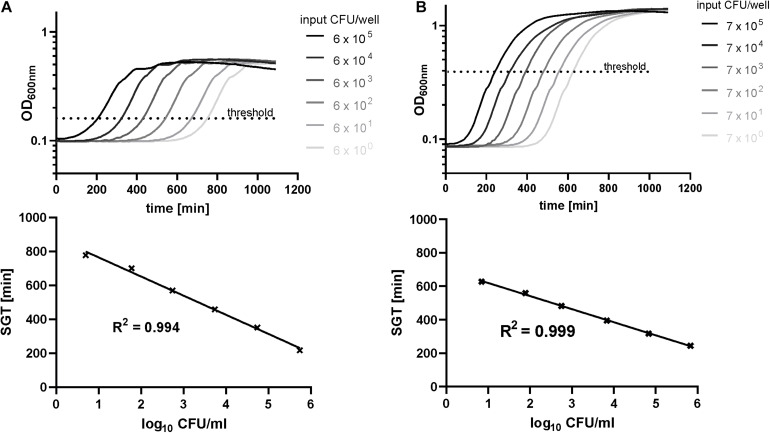
Standard curves determined by the SGT method and agar plating for exemplary isolates of *E. faecium* **(A)** and *S. aureus* **(B)** biofilms. The optical density at 600 nm was recorded for 18 h for a dilution series of resuspended biofilms (48 h of growth). Simultaneously, CFU numbers were determined by agar plating.

To verify the new high-throughput method from Hazan et al. (2012) for biofilm quantification, we recorded the growth curves of a dilution series of resuspended biofilms for each isolate. As on planktonic level time-lagged growth curves for biofilms could be observed in correlation to the CFU input (Figure 1). Comparable to a quantitative PCR, a specific OD threshold was defined within the midst of the exponential growth of the control biofilms. By this method, we received a linear correlation of SGT and CFU from 10^6^ to 10^0^ (Figure 1). The level of detection reached down to 2 CFU/well for both species whereby the level of detection and the detection range varied between experiments and isolates, especially for *E. faecium* (Supplementary Material).

**FIGURE 2:**
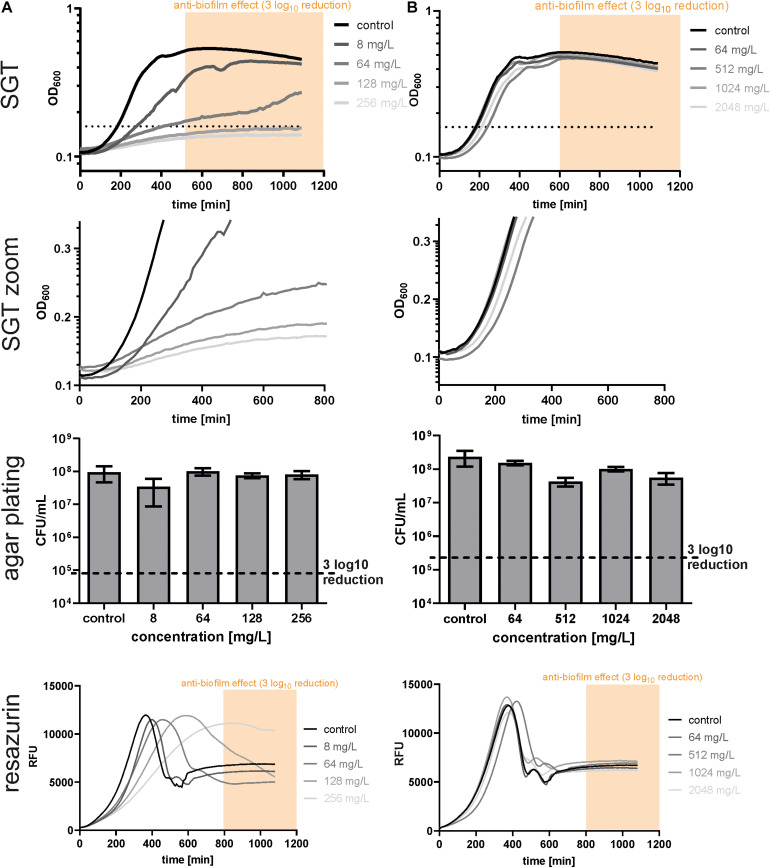
Determination of biofilm bactericidal effects with the three different methods in EF17129 biofilms treated with dalbavancin **(A)** and gentamicin **(B)**. The yellow or orange window (“anti-biofilm effect”) indicates the area with an at least 3 log_10_ reduction in CFU compared to the untreated control. The black dotted line indicates the OD threshold for SGT determination.

After calibration, we used the SGT method for analyzing the BBC of dalbavancin and gentamicin for *E. faecium* isolates (Figure 2). According to the SGT method, a 3 log_10_ CFU reduction was achieved at 128 mg/L of dalbavancin (Figure 2A). By contrast, agar plating revealed only a CFU reduction of < 1 log_10_ CFU at the highest dalbavancin concentration tested, thereby not achieving the required 3 log_10_ CFU reduction for the BBC (Figure 2A).

On closer inspection, it was striking that the regrowth of the former dalbavancin-treated biofilms started at the same time like the controls but had a slower growth kinetic (Figure 2A, zoom). This changed growth behavior might be due to a reduced metabolism after antibiotic treatment. Therefore, we checked our results by resazurin assays as described by Van den Driessche et al. (2014). In parallel to the SGT method, a standard curve was performed within the experiment to calculate the CFU to the respective resazurin t_*max*_ value (Supplementary Material). The BBC determined by the SGT method (BBC_*SGT*_) was consistent (± 1x BBC) with the BBC measured by the resazurin assay (BBC_*RESA*_) for dalbavancin at 256 mg/L (Figure 2A and Table 1).

**TABLE 1:** Comparison of biofilm bactericidal concentration (BBC) values obtained by different methods for biofilm quantification.

**Antibiotic**			**Calculated BBC (mg/L)**		
	**Strain**		**SGT (= BBC_*SGT*_)**	**Resazurin (= BBC_*RESA*_)**	**Agar plating (= BBC_*AGAR*_)**
Dalbavancin	*S. aureus*	4002	8	16	>32
		4733	4	2	>8
		1642/1	4	4	>8
	*E. faecium*	24498	8	8	>16
		12713	2	16	>64
		17129	128	256	>256
Rifampicin	*S. aureus*	4002	4	4	>8
		4733	0.125	0.125	>16
		1642/1	2	2	>8
Gentamicin	*E. faecium*	24498	64	64	64
		12713	128	128	128
		17129	>2,048	>2,048	>2,048

*The BBC is defined as the lowest concentration leading to 99.9% eradication of the biofilm (= 3 log_10_ CFU/mL reduction). SGT, Start-Growth-Time.*

For gentamicin-treated *E. faecium* biofilms, the SGT method revealed no anti-biofilm effect for all tested concentrations and showed no change in growth behavior (Figure 2B). The CFU determination by agar plating and resazurin obtained the same results (Figure 2B and Table 1). For the other two *E. faecium* isolates, BBC results obtained by all three methods were in accordance as well (Table 1 and Supplementary Material).

**FIGURE 3:**
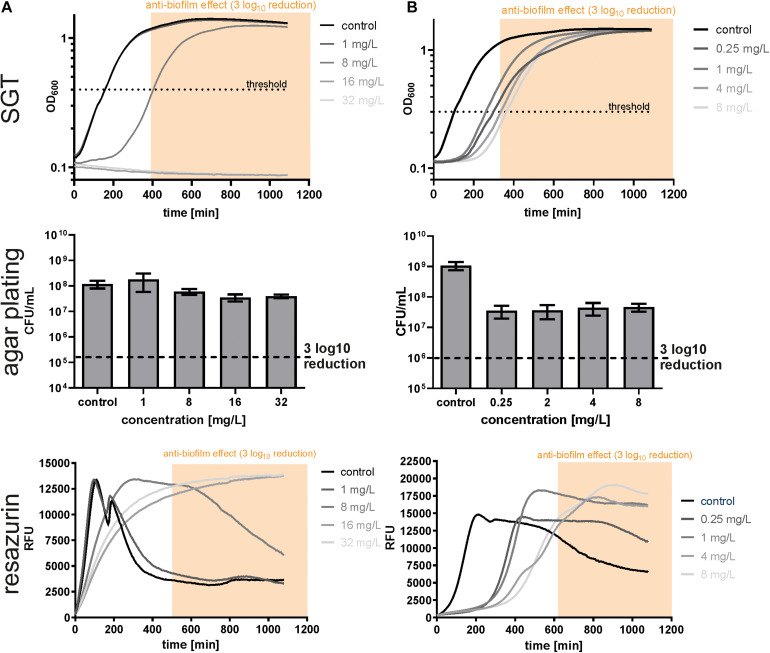
Determination of biofilm bactericidal effects with the three different methods in SA4002 biofilms treated with dalbavancin **(A)** and rifampicin **(B)**. The yellow window (“anti-biofilm effect”) indicates the area with an at least 3 log_10_ reduction in CFU compared to the untreated control.

To test whether the changed growth behavior observed in the SGT method is strain- or antibiotic-dependent, we analyzed *S. aureus* biofilms by all three methods using dalbavancin and rifampicin. In contrast to *E. faecium*, dalbavancin-treated biofilms showed normal time-lagged growth curves or no growth at all (Figure 3A). By the SGT method, we calculated a 3 log_10_ CFU reduction already at 8 mg/L while with agar plating there was nearly no CFU reduction detectable for all concentrations (Figure 3A). However, the BBC_*RESA*_ was again in line with the BBC_*SGT*_ (Figure 3A and Table 1). Similar results were seen for rifampicin-treated *S. aureus* biofilms (Figure 3B and Table 1). The BBC_*SGT*_ and BBC_*RESA*_ were both reached at 4 mg/L of rifampicin, whereas no BBC could be determined by agar plating since none of the tested rifampicin concentrations obtained a 3 log_10_ CFU reduction (Figure 3B).

## Discussion

In the presented study, the principle of the recently published, easy-to-use SGT-method was transferred from planktonic to biofilm-embedded cells, and compared to established, more labor-intensive methods. To integrate the SGT-method into the pool of already established methods for quantification of biofilm-embedded cells, biofilm bactericidal effects of three different antibiotics against mature *S. aureus* and *E. faecium* biofilms were measured by resazurin staining, agar plating and the SGT method. All methods are based on determination of CFU/mL values, either directly (agar plating) or indirectly via CFU/mL-calibrated standard curves (SGT, resazurin). While the SGT data correlated well with the results obtained by the resazurin assay, they only partially correlated with the results obtained by conventional agar plating. This led to a partial mismatch between the SGT/resazurin-derived BBCs (BBC_*SGT*_ and BBC_*RESA*_) and the current gold-standard, agar plating-derived BBCs (BBC_*AGAR*_), questioning the utility of the novel SGT-method—and of the well-established resazurin assay - for quantifying biofilm reducing effects.

Since gentamicin and rifampicin testing in contrast to dalbavancin testing resulted in comparable BBC values by all three methods, we arrived at the hypothesis that the SGT-method might only be well-suited for measuring the effect of certain antibiotic classes. We first thought about a classification into bactericidal/bacteriostatic antibiotics, but this categorization is usually based on planktonic cells and poorly transferable to biofilm-embedded cells (Pankey and Sabath, 2004). Biofilms resemble stationary-phase cultures with strongly reduced cell division rates, following that they are less susceptible to cell-wall active agents such as dalbavancin compared to planktonic cells, where cell wall active agents usually exhibit bactericidal effects. In contrast, the mode of actions of gentamicin and rifampicin do not require actively dividing cells since they target transcriptional and translational processes. Whether the mode of action of the antibiotics, i.e., whether they target growth-arrested or actively dividing cells, influences the utility of the SGT method for determining biofilm reducing effects requires further testing.

Another possible explanation for the discrepancy between BBC_*SGT*_ and BBC_*AGAR*_ values might be due to the method-related comparison of two different time-points of bacterial growth. While the agar plating method assesses the number of CFUs in the stationary phase after 24 h of growth, the SGT method pictures the regrowth of the former biofilm-embedded cells over time, with the SGT values theoretically being calculated in the midst of the logarithmic phase. However, instead of being parallel and lagged to the untreated growth control as seen for gentamicin treatment, some antibiotic treatment-derived growth curves showed a significantly slower increase with reduced cell doubling times and without distinct growth phases, as seen for dalbavancin-treated *E. faecium* biofilms. Here, the reduced growth kinetics of the cells led to higher SGT values since the OD_600 *nm*_ threshold was reached later. As a consequence, the higher SGT values were falsely interpreted as lower number of cells leading to an overestimation of antibiotic bactericidal efficacy by the SGT method. To analyze whether the recovery from antibiotic-induced stress is also accompanied by a decreased CFU development on agar plates, we checked the number of CFUs formed on agar plates in 1 h intervals for a period of 14 h. The hypothesis hereby was that the colonies of growth kinetic-altered samples appear later and might show an altered phenotype, e.g., formation of small colony variants, but finally result in the same number of cells as the untreated control. However, no difference in the time point of CFU appearance or shape, size and color could be observed between treated and untreated samples (data not shown). Changes in colony formation were generally hard to depict though since both *E. faecium* and *S. aureus* form relatively small colonies.

As indicated by high SGT and low resazurin values, dalbavancin seemed to be able to slow down the bacterial metabolism, thereby reducing the redox potential of the cells and changing the growth behavior. However, these cells were still cultivable as reflected by unchanged CFU_*AGAR*_ numbers. For *E. faecium* EF17129 no effect of dalbavancin was expected since this isolate shows a vanA-VRE genotype, which exhibits high-level resistance to dalbavancin (Biedenbach et al., 2009). Bacteria use different strategies for survival during exposure to antibiotics, namely resistance, tolerance and persistence (Brauner et al., 2016). Resistance describes the inherited ability of bacteria to grow, i.e., to proliferate, at high concentrations of an antibiotic irrespective of the duration of treatment due to gene mutations. In contrast, tolerant cells survive high antibiotic concentrations by transiently slowing down essential bacterial processes at the cost of loss of cell proliferation. Once the transient trigger for tolerance is removed, cells do recover and growth can continue. While resistance and tolerance are attributes of the whole bacterial population, persistence is only attributable to a subpopulation (typically around 1%) of clonal cells. Persistent cells can survive at high concentrations of antibiotics whereas the majority of the clonal bacterial population is rapidly killed (Lewis, 2010). While antibiotic-resistant bacteria can form biofilms, the survival strategies characteristic for biofilms are antibiotic tolerance and persister cell formation (Stewart, 2002). We therefore hypothesized that the altered growth kinetics observed in growth curves of dalbavancin-treated biofilms were caused by the physiological rearrangements necessary to leave the tolerant state once the antibiotic had been removed and the biofilms were re-transferred to the planktonic phase. To test this hypothesis, we performed time-kill measurements of biofilm-derived planktonic cells (i.e., cells which have been grown into biofilms, have been treated and then were re-transferred into the planktonic phase for recording of the growth curves) versus non-biofilm, conventional planktonic cells treated with 20x MIC_*DALBAVANCIN*_ to determine the so-called minimal duration of killing (MDK). The MDK_99__%_ describes the time amount needed to kill 99% of the bacterial population and is derived by plotting viable CFUs against time (Hazan et al., 2014; Brauner et al., 2016). While the MIC is used as a standardized metric to measure antibiotic resistance, the MDK has been suggested as a metric for measurement of tolerance and persistence. If truly tolerant cells had emerged due to treatment of the biofilms, a higher MDK_99__%_ would have been expected for the biofilm-derived planktonic cells compared to the non-biofilm, conventional planktonic cells. However, no difference in the MDK_99__%_ was observed for both cell types indicating no physiological differences and no tolerance effects (data not shown).

A further explanation for the altered growth kinetics of dalbavancin-treated biofilms might be the transient uptake of glycopeptide molecules in the cell wall of Gram-positive organisms, as described for vancomycin-intermediate *S. aureus*. Vancomycin molecules were shown to bind not only to their main target, namely d-alanyl-d-alanine residues of cell wall precursors, but also to excess free d-alanyl-d-alanine residues randomly distributed in the cell wall of *S. aureus* (Cui et al., 2006). These second targets of vancomycin lead to clogging and cell wall thickening, preventing vancomycin to reach its true target at the growing peptidoglycan chain, thus mediating resistance (Cui et al., 2003). Since dalbavancin as a glycopeptide targets d-alanyl-d-alanine residues as well, a similar mechanism where *E. faecium* absorbs single dalbavancin molecules in its cell wall is possible (Zhanel et al., 2010). Upon transfer from the biofilm to the planktonic phase in the SGT method processing, the cell wall-residing dalbavancin molecules might be released again in the media due to renewed cell division, leading to delayed growth and altered growth kinetics. To verify this hypothesis, we performed again a SGT measurement with *E. faecium* and *S. aureus* biofilms after dalbavancin treatment (Supplementary Figure 9). To check for dalbavancin residues in the biofilm as well as in the cell wall, we collected the supernatant of the resuspended biofilm cells at different time points during their cell growth. After collection, standardized amounts of fresh bacterial cells were added to each supernatant and possible growth-inhibitory effects were analyzed similar to MIC testing. For *E. faecium*, a significant growth delay was only observed for the cells which had been treated with the supernatant taken from 128 mg/L dalbavancin treatment at time point 0 h, indicating the release of sufficient dalbavancin directly out of the biofilm upon resuspension to interfere with bacterial growth (Supplementary Figure 9A). No change in growth was observed for the cells treated with the other supernatants, implicating no release of dalbavancin molecules out of the cell wall. In contrast, experiments with *S. aureus* indicated the release of dalbavancin of both, the biofilm and the cell wall, confirming above hypothesis (Supplementary Figure 9B). Since for agar plating only high dilutions were evaluated to allow for CFU counting, released dalbavancin molecules were diluted as well, therefore no change in CFU numbers or cell growth was seen although the same effect is likely present. Importantly, the adherence of dalbavancin to the biofilm and the cell wall does not only lead to false positive anti-biofilm effects with the SGT method (and consequently to a mismatch between BBC_*SGT*_ and BBC_*AGAR*_) but also with the resazurin method. Resazurin is a stable redox indicator which’s highly fluorescent reduction product resorufin can be easily and rapidly measured after 30–120 min of cell contact and is proportional to the number of metabolically active cells (Azeredo et al., 2017). Since the linear range between resorufin and CFU numbers is restricted to 10^6^–10^8^ CFU/mL, the conventional resazurin-based viability assay fails to depict a 3 log_10_ reduction required for BBC calculation (Sandberg et al., 2009). We therefore used a recently published optimized method determining the time needed to reach the maximum fluorescence extending the linear range to 10^3^–10^8^ CFU/mL (Van den Driessche et al., 2014). While this new approach claims to accurately reflect CFU numbers as determined by agar plating, our results indicate that this was not true for dalbavancin. Here, dalbavancin residues in the biofilm interfered as well with the actual methodology resulting in falsely lowered metabolic activity and therefore overestimation of the anti-biofilm effect. Since resazurin is being increasingly used to study microbial biofilms (Azeredo et al., 2017), researchers should be aware of a potential correlation bias for some antibiotics.

In conclusion, the adherence of dalbavancin to the biofilm and cell wall led to false positive anti-biofilm effects with the SGT method, since the altered growth kinetics and consequential high SGT values were not due to the initial treatment of the biofilm but due to a renewed antibiotic challenge of the biofilm-resuspended planktonic cells during regrowth. The application of the SGT method for anti-biofilm testing is therefore not suited for antibiotics which stick to the biofilm and/or to the cell wall. Since it remains unknown for which antibiotic-biofilm combinations such effects occur, a prior testing before high-throughput application of the SGT method for measurement of CFU reduction is mandatory for anti-biofilm testing. It is important to note that we adapted the SGT method for Gram-positive biofilms only; it remains unclear whether Gram-negative biofilms show the same tendency to transiently uptake glycopeptide molecules in the biofilm matrix and/or in the cell wall as observed for Gram-positive biofilms in this study. Further studies are necessary to adapt the SGT method to Gram-negative biofilms and to find a solution for counteracting the adherence effect of dalbavancin to the biofilm, i.e., to avoid an unintended, renewed antibiotic challenge. However, if a comparison of CFU_*SGT*_ with CFU_*AGAR*_ for a specific antibiotic exhibits a good correlation, CFU_*SGT*_ as a much less labor-intensive method may be used for high-throughput screening as required for microbiological routine testing.

## Data Availability Statement

The original contributions presented in the study are included in the article/Supplementary Material, further inquiries can be directed to the corresponding author/s.

## Author Contributions

LT conceptualized the article, designed, performed the experiments, analyzed, interpreted the data, wrote, and revised the manuscript. AH designed, performed the experiments, analyzed, interpreted the data, and wrote the methods and results part of the manuscript. KT, JG, and RS revised and critically discussed the article. OM and MP revised the manuscript for important intellectual content. All authors read and approved the final manuscript.

## Conflict of Interest

The authors declare that the research was conducted in the absence of any commercial or financial relationships that could be construed as a potential conflict of interest.

## Publisher’s Note

All claims expressed in this article are solely those of the authors and do not necessarily represent those of their affiliated organizations, or those of the publisher, the editors and the reviewers. Any product that may be evaluated in this article, or claim that may be made by its manufacturer, is not guaranteed or endorsed by the publisher.

## References

[B1] Azeredo J. Azevedo N. F. Briandet R. Cerca N. Coenye T. Costa A. R. (2017). Critical review on biofilm methods. *Crit. Rev. Microbiol.* 43 313–351. 27868469 10.1080/1040841X.2016.1208146

[B2] Biedenbach D. J. Bell J. M. Sader H. S. Turnidge J. D. Jones R. N. (2009). Activities of dalbavancin against a worldwide collection of 81,673 gram-positive bacterial isolates. *Antimicrob. Agents chemother.* 53 1260–1263. 10.1128/aac.01453-08 19124664 PMC2650578

[B3] Brauner A. Fridman O. Gefen O. Balaban N. Q. (2016). Distinguishing between resistance, tolerance and persistence to antibiotic treatment. *Nat. Rev. Microbiol.* 14 320–330. 10.1038/nrmicro.2016.34 27080241

[B4] Coenye T. Goeres D. Van Bambeke F. Bjarnsholt T. (2018). Should standardized susceptibility testing for microbial biofilms be introduced in clinical practice? *Clin. Microbiol. Infect.* 24 570–572. 10.1016/j.cmi.2018.01.003 29337253

[B5] Coraça-Huber D. C. Fille M. Hausdorfer J. Pfaller K. Nogler M. (2012). *Staphylococcus aureus* biofilm formation and antibiotic susceptibility tests on polystyrene and metal surfaces. *J. Appl. Microbiol.* 112 1235–1243. 10.1111/j.1365-2672.2012.05288.x 22435667

[B6] Cruz C. D. Shah S. Tammela P. (2018). Defining conditions for biofilm inhibition and eradication assays for Gram-positive clinical reference strains. *BMC Microbiol.* 18:173. 10.1186/s12866-018-1321-6 30390625 PMC6215609

[B7] Cui L. Iwamoto A. Lian J. Q. Neoh H. M. Maruyama T. Horikawa Y. (2006). Novel mechanism of antibiotic resistance originating in vancomycin-intermediate Staphylococcus aureus. *Antimicrob. Agents Chemother.* 50 428–438. 10.1128/aac.50.2.428-438.2006 16436693 PMC1366884

[B8] Cui L. Ma X. Sato K. Okuma K. Tenover F. C. Mamizuka E. M. (2003). Cell wall thickening is a common feature of vancomycin resistance in *Staphylococcus aureus*. *J. Clin. Microbiol.* 41 5–14. 10.1128/jcm.41.1.5-14.2003 12517819 PMC149586

[B9] Flemming H. C. Wingender J. Szewzyk U. Steinberg P. Rice S. A. Kjelleberg S. (2016). Biofilms: an emergent form of bacterial life. *Nat. Rev. Microbiol.* 14 563–575. 10.1038/nrmicro.2016.94 27510863

[B10] Hall-Stoodley L. Stoodley P. Kathju S. Høiby N. Moser C. Costerton J. W. (2012). Towards diagnostic guidelines for biofilm-associated infections. *FEMS Immunol. Med. Microbiol.* 65 127–145. 10.1111/j.1574-695x.2012.00968.x 22469292

[B11] Hazan R. Maura D. Que Y. A. Rahme L. G. (2014). Assessing *Pseudomonas aeruginosa* Persister/antibiotic tolerant cells. *Methods Mol. Biol.* 1149 699–707. 10.1007/978-1-4939-0473-0_54 24818944 PMC6538066

[B12] Hazan R. Que Y. A. Maura D. Rahme L. G. (2012). A method for high throughput determination of viable bacteria cell counts in 96-well plates. *BMC Microbiol.* 12:259. 10.1186/1471-2180-12-259 23148795 PMC3534621

[B13] Lewis K. P. (2010). Persister cells. *Annu. Rev. microbiol.* 64 357–372. 20528688 10.1146/annurev.micro.112408.134306

[B14] Macià M. D. Rojo-Molinero E. Oliver A. (2014). Antimicrobial susceptibility testing in biofilm-growing bacteria. *Clin. Microbiol. Infect.* 20 981–990. 10.1111/1469-0691.12651 24766583

[B15] Magana M. Sereti C. Ioannidis A. Mitchell C. A. Ball A. R. Magiorkinis E. (2018). Options and limitations in clinical investigation of bacterial biofilms. *Clin. Microbiol. Rev.* 31 e00084–16. 10.1128/CMR.00084-16 PMC6056845 29618576

[B16] Neudorfer K. Schmidt-Malan S. M. Patel R. (2018). Dalbavancin is active in vitro against biofilms formed by dalbavancin-susceptible enterococci. *Diagn. Microbiol. Infect. Dis.* 90 58–63. 10.1016/j.diagmicrobio.2017.09.015 29195766

[B17] Pankey G. A. Sabath L. D. (2004). Clinical relevance of bacteriostatic versus bactericidal mechanisms of action in the treatment of Gram-positive bacterial infections. *Clin. Infect. Dis.* 38 864–870. 10.1086/381972 14999632

[B18] Sandberg M. E. Schellmann D. Brunhofer G. Erker T. Busygin I. Leino R. (2009). Pros and cons of using resazurin staining for quantification of viable *Staphylococcus aureus* biofilms in a screening assay. *J. Microbiol. Methods* 78 104–106. 10.1016/j.mimet.2009.04.014 19427338

[B19] Sandoe J. A. Wysome J. West A. P. Heritage J. Wilcox M. H. (2006). Measurement of ampicillin, vancomycin, linezolid and gentamicin activity against enterococcal biofilms. *J. Antimicrob. Chemother.* 57 767–770. 10.1093/jac/dkl013 16464896

[B20] Stewart P. S. (2002). Mechanisms of antibiotic resistance in bacterial biofilms. *Int. J. Med. Microbiol.* 292 107–113. 10.1078/1438-4221-00196 12195733

[B21] Thieme L. Hartung A. Tramm K. Klinger-Strobel M. Jandt K. D. Makarewicz O. (2019). MBEC versus MBIC: the lack of differentiation between biofilm reducing and inhibitory effects as a current problem in biofilm methodology. *Biol. Proced. Online* 21:18. 31528123 10.1186/s12575-019-0106-0 PMC6743098

[B22] Van den Driessche F. Rigole P. Brackman G. Coenye T. (2014). Optimization of resazurin-based viability staining for quantification of microbial biofilms. *J. Microbiol. Methods* 98 31–34. 10.1016/j.mimet.2013.12.011 24365109

[B23] Zhanel G. G. Calic D. Schweizer F. Zelenitsky S. Adam H. Lagacé-Wiens P. R. (2010). New lipoglycopeptides: a comparative review of dalbavancin, oritavancin and telavancin. *Drugs* 70 859–886. 10.2165/11534440-000000000-00000 20426497

[B24] Zimmerli W. Sendi P. (2019). Role of rifampin against staphylococcal biofilm infections in vitro, in animal models, and in orthopedic-device-related infections. *Antimicrob. Agents Chemother.* 63 e01746–18. 30455229 10.1128/AAC.01746-18 PMC6355554

