# Supplementary material for: Adaptation of the Start-Growth-Time Method for High-Throughput Biofilm Quantification
Source: Front Microbiol. 2021 Aug 26;12:631248. doi: 10.3389/fmicb.2021.631248 (PMC8428173; doi:10.3389/fmicb.2021.631248)
Supplement: Supplementary file 1 [file Data_Sheet_1.docx]

Supplementary Material


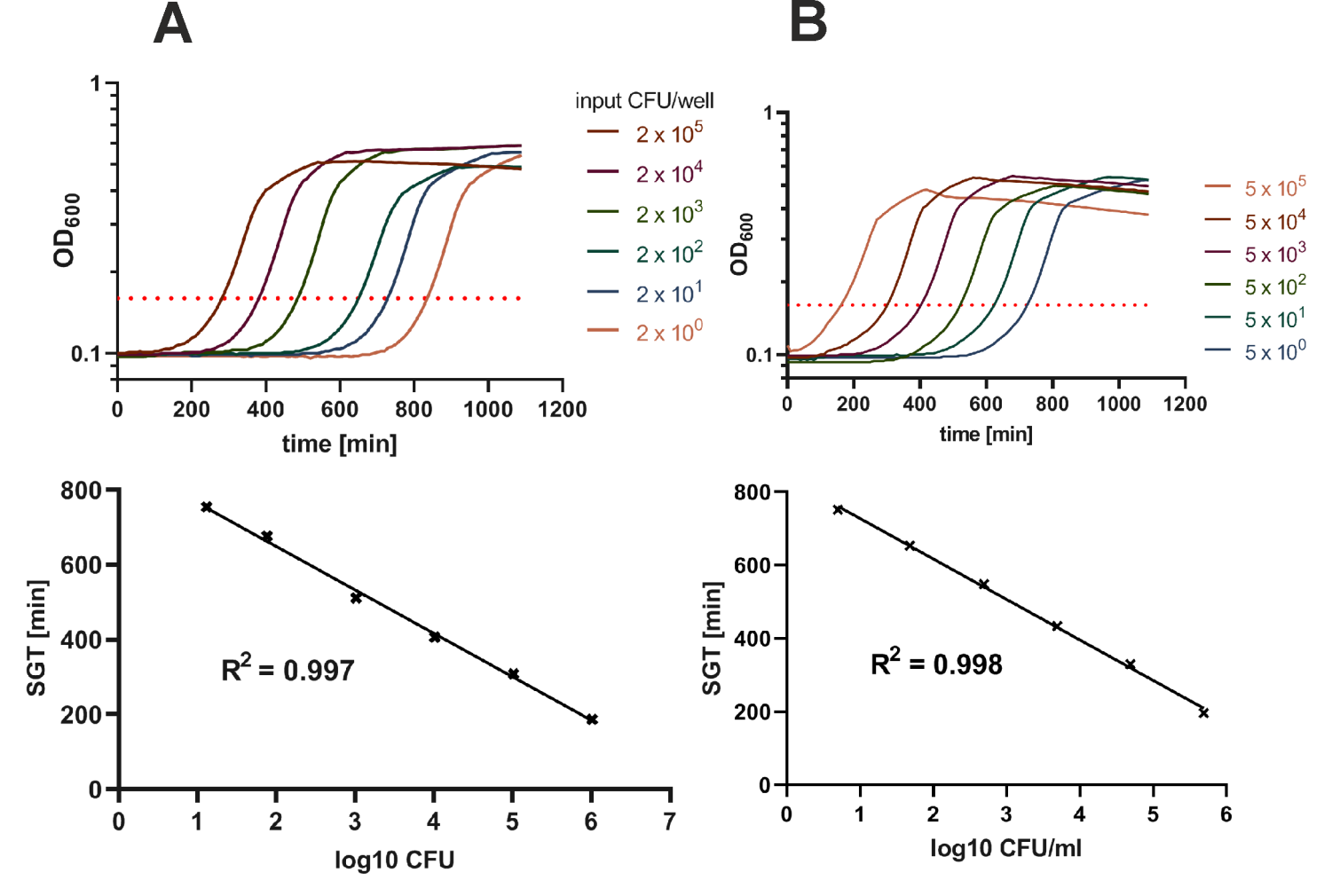
**Figure S1**: SGT standard curves for the other two *E. faecium* isolates, EF24498 **(A)** and EF12713 **(B)**. The red dotted line indicates the threshold for SGT determination.


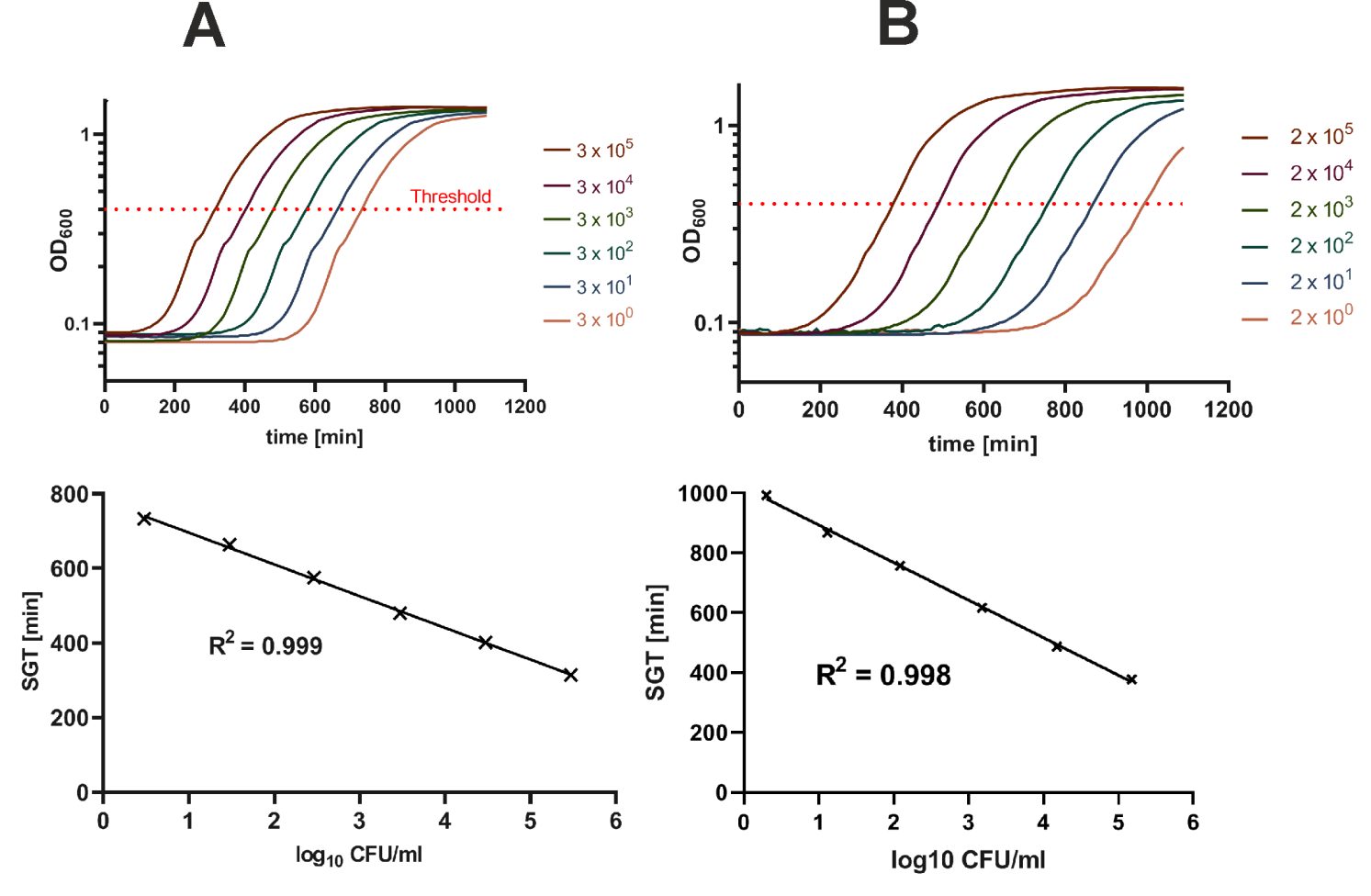
**Figure S2**: SGT standard curves for the residual *S. aureus* isolates, SA4733 **(A)** and SA1642 **(B)**. The red dotted line indicates the threshold for SGT determination.


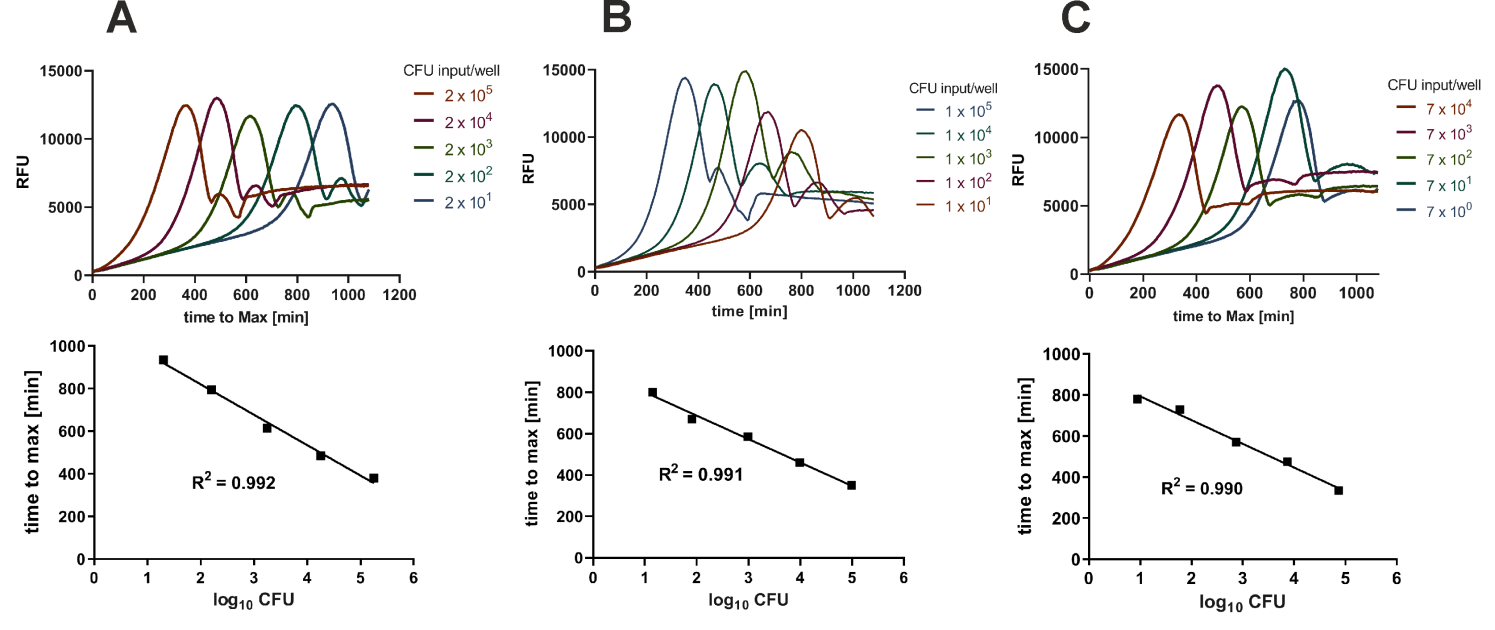
**Figure S3**: Resazurin standard curves for *E. faecium* EF17129 **(A)**, EF24498 **(B)** and EF12713 **(C)**. Analysis of resazurin assay was done by time to RFU maximum determination.


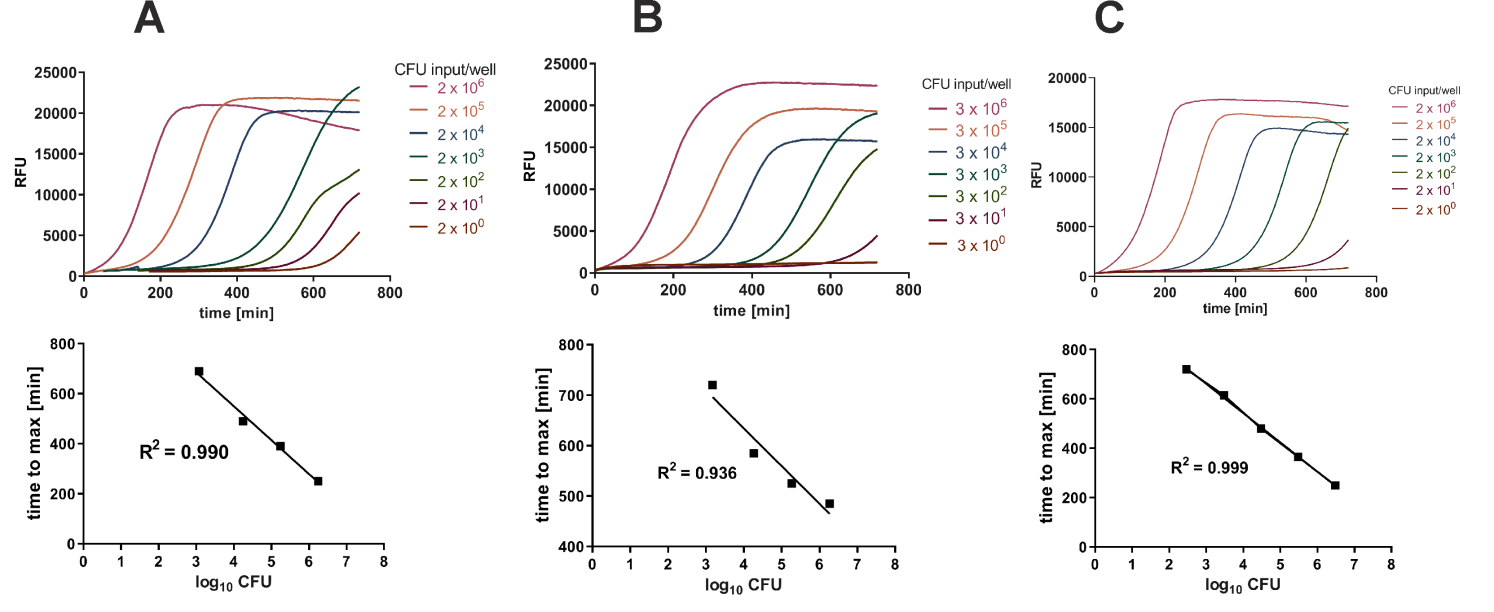
**Figure S4**: Resazurin standard curves for *S. aureus* SA4002 **(A)**, SA4733 **(B)** and SA1642 **(C)**. Analysis of resazurin assay was done by time to RFU maximum determination.


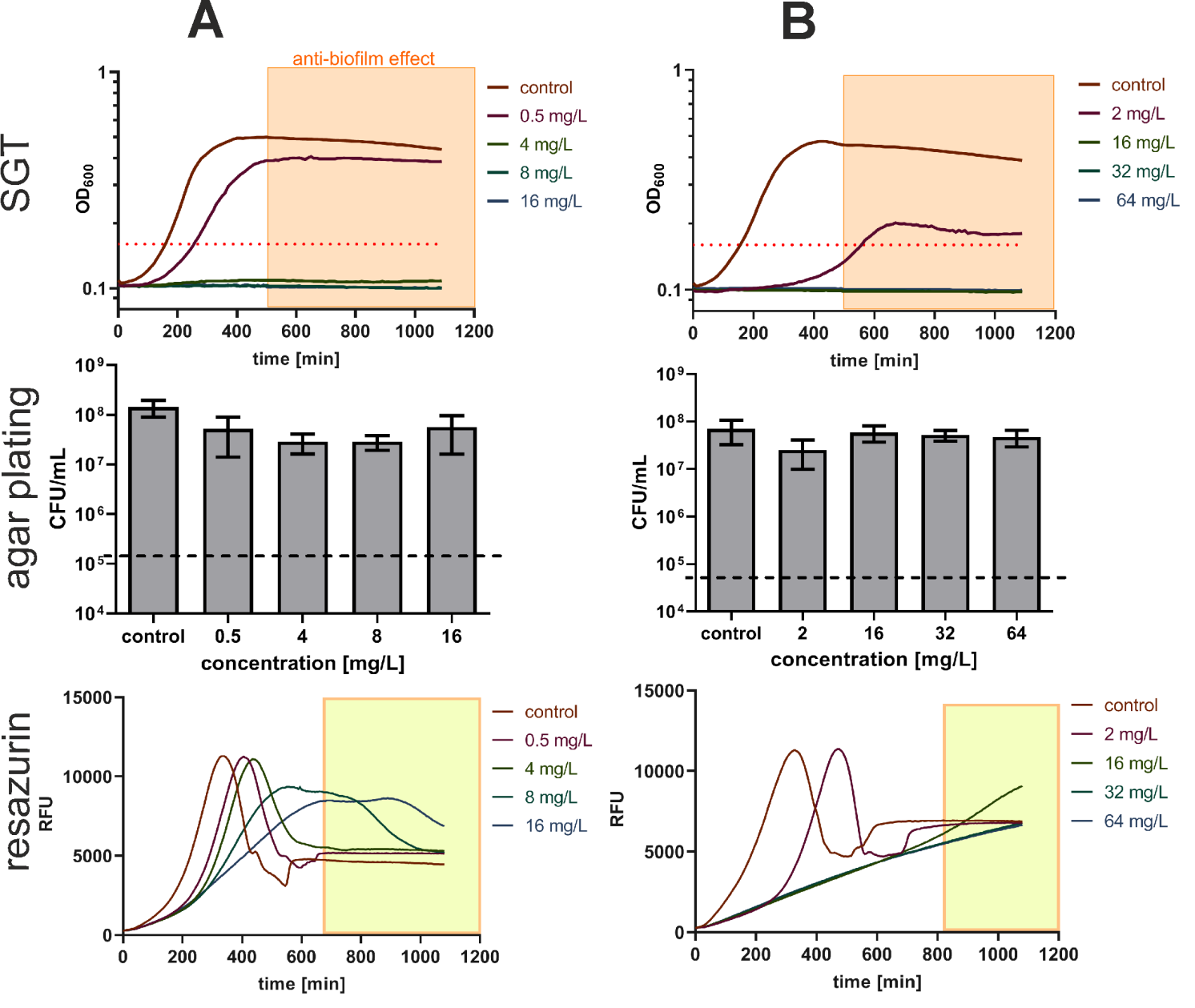
**Figure S5**: Analysis of dalbavancin treated *E. faecium* biofilms by SGT, agar plating and resazurin assay for EF24498 **(A)** and EF12713 **(B)**. The yellow and orange windows indicate a >3 log_10_ CFU reduction compared to the untreated control. The red dotted line indicates the threshold for SGT determination.


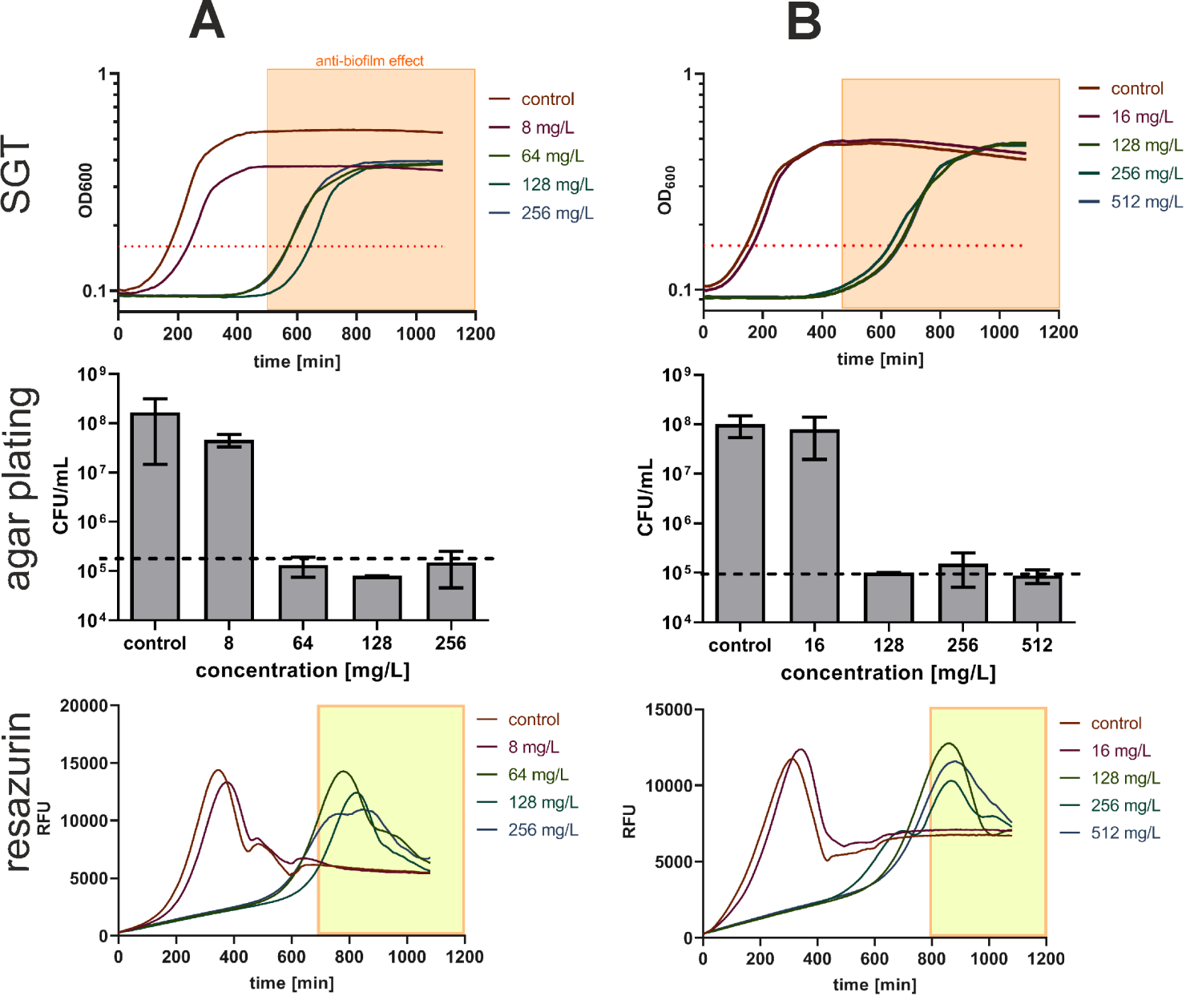
**Figure S6**: Analysis of gentamicin treated *E. faecium* biofilms by the three different methods for EF24498 **(A)** and EF12713 **(B)**. The orange and yellow windows indicate a >3 log_10_ CFU reduction compared to untreated control. The red dotted line indicates the threshold.


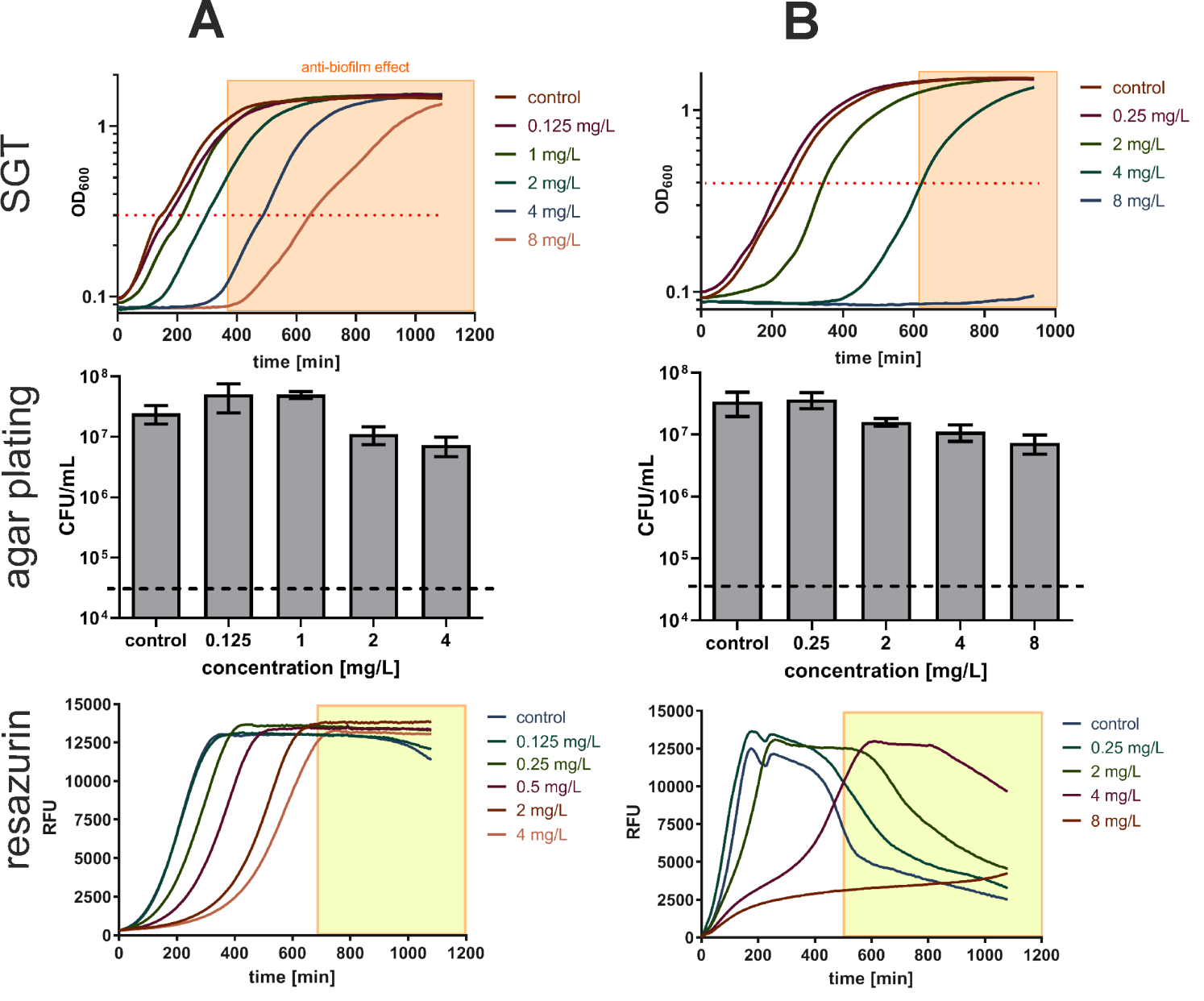
**Figure S7**: Analysis of dalbavancin treated *S.aureus* biofilms by SGT, agar plating and resazurin assay for SA4733 **(A)** and SA1642 **(B)**. The orange and yellow windows indicate a >3 log_10_ CFU reduction compared to untreated control. The red dotted line indicates the threshold.


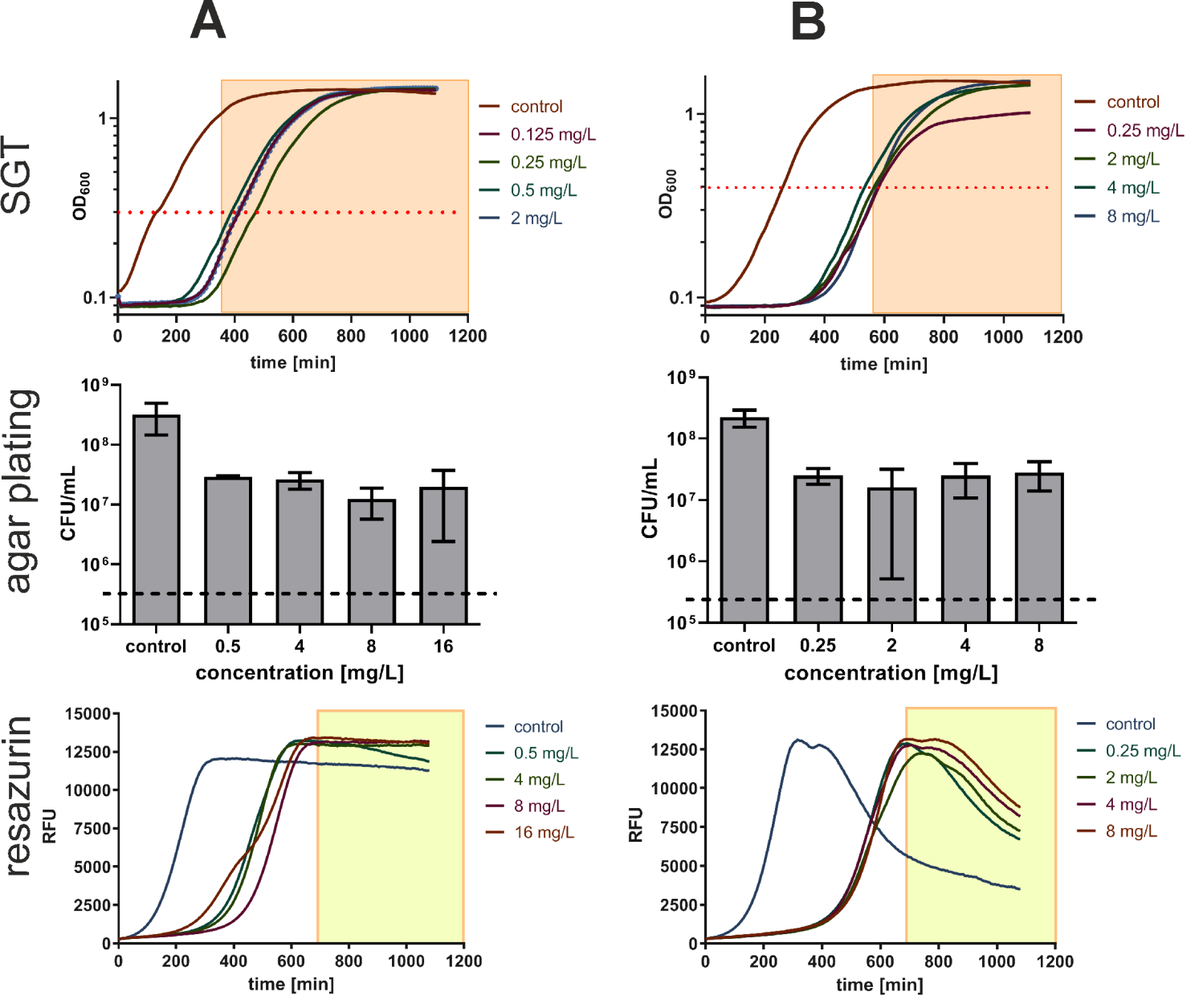
**Figure S8**: Analysis of rifampicin treated *S. aureus* biofilms by the three different methods for SA4733 **(A)** and SA1642 **(B)**. The yellow and orange windows indicate a >3 log_10_ CFU reduction compared to untreated control. The red dotted line indicates the threshold.


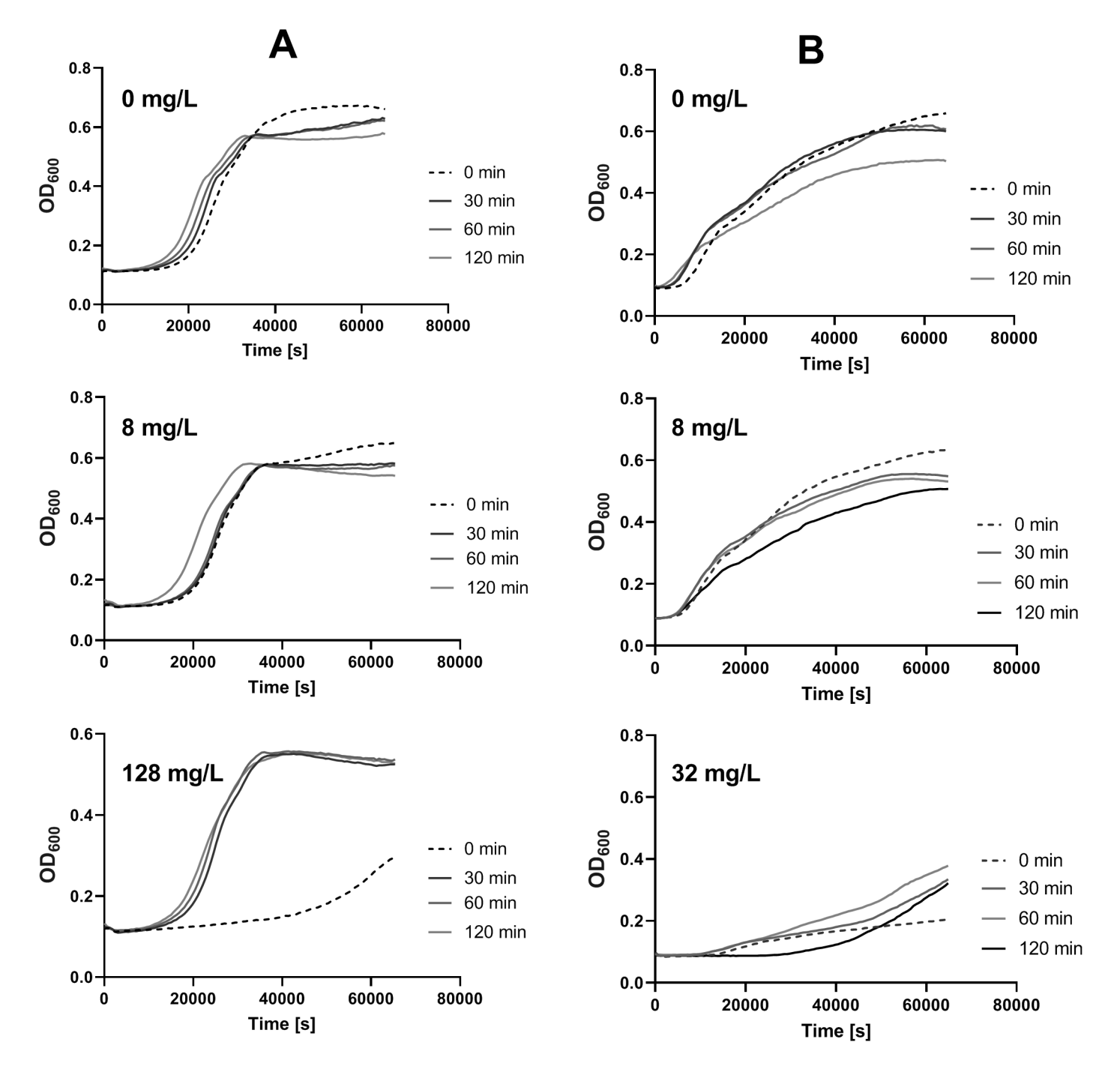


Figure S9: Growth curves for determination of dalbavancin release after disruption of biofilms EF17129 (Column A) and SA4002 (Column B). To test whether the delayed growth seen in the SGT is due to residues of dalbavancin in the growth media, we mixed fresh planktonic cells and the supernatant of resuspended biofilms treated with 0, 8 and 128 mg/L (*E. faecium*) or 0,8 and 32 mg/L dalbavancin (*S. aureus)*. Therefore, in contrast to the normal SGT analysis, resuspended biofilms were centrifugated. The supernatant was collected to determine the antibiotic release directly from the biofilm (time point 0 min). The cell pellet was washed once with 0.9% sodium chloride to remove all possible antibiotic residues. Cells were then resuspended and grown in fresh media. After 30, 60 and 120 minutes, an aliquot of the culture was centrifugated and supernatant was collected to check if any cell-wall bound antibiotic was released to the media. All collected supernatants from the four time points were mixed with 10^6^ CFU/mL (according to MIC testing) and OD was recorded at 600 nm over 18 h in a microplate reader (Sunrise, Tecan, Switzerland). Interestingly, by disruption/scraping of the biofilms treated with the highest concentrations of dalbavancin slowed down growth was observed for EF17129 (directly after Biofilm disruption = 0 min) and for SA4002 at all time points.
